# Prognostic Value of Pre-Treatment [18F]FDG PET/CT Texture Analysis in Undifferentiated Soft-Tissue Sarcoma

**DOI:** 10.3390/jcm12010279

**Published:** 2022-12-29

**Authors:** Alessio Annovazzi, Virginia Ferraresi, Renato Covello, Andrea Ascione, Sabrina Vari, Maria Grazia Petrongari, Jacopo Baldi, Roberto Biagini, Rosa Sciuto

**Affiliations:** 1 Nuclear Medicine Unit, IRCCS—Regina Elena National Cancer Institute, 00144 Rome, Italy; 2 Sarcomas and Rare Tumors Departmental Unit, IRCCS—Regina Elena National Cancer Institute, 00144 Rome, Italy; 3 Department of Pathology, IRCCS—Regina Elena National Cancer Institute, 00144 Rome, Italy; 4 Department of Radiological, Oncological and Pathological Sciences, Sapienza University of Rome, 00161 Rome, Italy; 5 Department of Radiation Oncology, IRCCS—Regina Elena National Cancer Institute, 00144 Rome, Italy; 6 Oncological Orthopaedics Unit, IRCCS—Regina Elena National Cancer Institute, 00144 Rome, Italy

**Keywords:** 18F-FDG, PET/CT, undifferentiated soft-tissue sarcoma, predictive value, radiomics

## Abstract

Background: Undifferentiated soft-tissue sarcomas (USTS) are one of the most common sarcoma histotypes in adults. The standard of care is surgical excision plus adjuvant radiotherapy, while the use of perioperative chemotherapy is still controversial. The aim of this study was to investigate the value of pre-treatment [18F]FDG PET/CT conventional metrics and textural features in predicting disease-free survival (DFS) and overall survival (OS) in patients with USTS of the limbs and trunk. Methods: [18F]FDG PET/CT scans of 51 consecutive patients with locally advanced USTS were retrospectively evaluated. Conventional and textural PET parameters were analysed and tested as predictive factors for DFS and OS. Results: During a median follow up of 50.7 months, 23 (45.1%) and 29 (56.9%) patients had death or disease progression, respectively. Univariate analysis revealed a significant association for perioperative treatment, PET volumetric parameters and the textural feature GLCM_correlation with DFS and OS. In multivariate analysis, perioperative treatment and GLCM_correlation were the only independent factors, allowing stratification of the population into three different prognostic classes. Conclusion: GLCM_correlation can identify USTS at high risk of relapse and death, thus helping to optimize the perioperative treatment of patients.

## 1. Introduction

Soft-tissue sarcomas (STS) represent a heterogeneous class of rare tumours with mesenchymal differentiation, which include more than 80 different histologic subtypes with variable clinical and biological behaviour, accounting for 1% of all adult malignancies [1,2].

Undifferentiated soft-tissue sarcoma (USTS) is the third most common histotype in adults after leiomyosarcoma and liposarcoma. Unlike other histotypes, USTS lacks immunohistochemical markers for a specific cell differentiation, thus representing a diagnosis of exclusion. It usually arises in the deep tissue of the lower extremities and presents aggressive behaviour with a local recurrence and metastatic rate of 30–35%, generally occurring 12 to 24 months after diagnosis [3]. The standard of care is en bloc surgical excision with R0 margins, which should be carried out by a specifically trained oncologic surgeon. Radiotherapy (RT) is usually added to surgery for stage II, IIIA and IIIB [4], but its use in the preoperative setting is on the rise.

The use of neoadjuvant or adjuvant chemotherapy is, however, still controversial [5], even though USTS represents one of the most responsive histotypes [6]. For this reason, the identification of potential parameters assessing the biological aggressiveness of the neoplasm could be crucial in adopting the most effective therapeutic strategy. The [18F]FDG PET/CT approach is increasingly used in the staging and re-staging of STS, and it has been shown to influence their management [7,8]. However, [18F]FDG PET/CT is also emerging as a possible prognostic tool in soft-tissue sarcomas (STS). The standardized uptake maximum value (SUVmax) has been reported to correlate with overall survival (OS) in mixed cohorts of STS [9,10], but also in a specific histological subgroup of synovial sarcoma [11]. Several studies have suggested that volume-based PET parameters, such as Metabolic Tumor Volume (MTV) and Tumor Lesion Glycolysis (TLG), are superior for predicting outcome in STS , providing more insight into whole tumor metabolism than a single maximum voxel value [12,13], although this finding has not been confirmed in all the studies [10,14]. The correlation between the above PET parameters with survival in STS was also reported in a meta-analysis by Chen et al. [15]. However, all these works refer to mixed populations of patients with sarcomas also including bone sarcomas and low grade neoplasms.

The aim of the present study was to investigate the value of standard and textural features of pre-treatment [18F]FDG PET/CT in predicting disease-free survival (DFS) and overall survival (OS) in patients with localized, resectable USTS. We further aimed to assess the correlation of histologic features with PET parameters.

## 2. Materials and Methods

### 2.1. Patients

**Figure 1:**
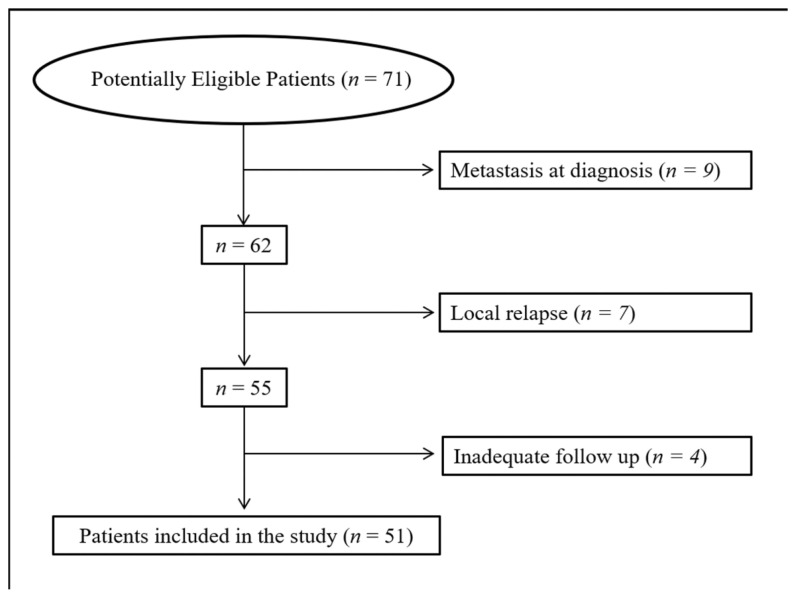
Flow chart of the patient selection process.

The study was approved by the local institutional ethics committee (Prot. no. 1679/22) and was performed in compliance with the ethical standards. Given the retrospective design of the study, the ethics committee allowed the use and processing of the patient clinical data, even in the absence of written informed consent. A database search was performed for patients with primary locally advanced non-metastatic USTS, operated on at our Institute between June 2012 and December 2020, who underwent an [18F]FDG PET/CT before surgery or neoadjuvant treatment. As the aim of the study was to investigate the role of [18F]FDG PET/CT in predicting DFS and OS in patients with USTS at first diagnosis, patients with local recurrence (*n* = 7) and those with available follow-up <6 months (*n* = 4) were excluded from the analysis. A flowchart of the patient selection process is shown in Figure 1. A final cohort of 51 patients was finally analysed. Patient clinical outcome was measured by disease-free survival (DFS) and overall survival (OS), defined as the period starting from the date of first treatment and disease relapse or death, respectively. The indication for a possible perioperative treatment, both in terms of timing (neoadjuvant and/or adjuvant) and type of therapy (radiotherapy alone, chemotherapy + radiotherapy, chemotherapy alone), was given by the sarcoma disease management team of our institute according to current guidelines [16], taking into account the morbidity of the treatment, as well as any comorbidities, life expectancy and the patients’ wishes.

### 2.2. Pathologic Evaluation

The histologic diagnosis of USTS was made on surgical specimens in accordance with the 2020 WHO classification of bone and soft-tissue sarcomas [1]. All available slides for each case were reviewed by AA, a pathologist in training with 3 years of experience, and RC, a pathologist with more than 25 years of experience in the field of soft-tissue tumours. Each case was evaluated independently by the two readers for a series of histopathological features, with a joint session to settle disagreements. USTS were classified as pleomorphic in 44 cases and spindle cell in 7; no round-cell tumors were encountered in this series. To exclude a specific line of differentiation, tumors were analysed by immunohistochemistry and, in selected cases, also by “Next Generation Sequencing” (NGS). Surgical margins were classified in accordance with Enneking staging (intralesional, marginal, wide, radical); all specimens analysed showed adequate (wide or radical) margins. The mitotic index was calculated as the number of mitotic figures present in 10 consecutive high-power fields (HPF) in the most mitotically active area of the tumour. Atypical mitotic figures and apoptotic figures were evaluated as absent, rare (<3 per 10 HPF) or diffuse (≥3 per 10 HPF). Pleomorphism was listed as mild, moderate or severe. Necrosis was reported as a percentage, considering all the available slides per case. Cellularity was calculated as the percentage of the tumour area occupied by neoplastic cells. The inflammatory infiltrate was graded as absent, mild, moderate or intense, and classified according to its nature (e.g., lymphocytic, mixed, giant cell-rich, eosinophilic). The French Federation Nationale des Centres de Lutte Contre le Cancer (FNCLCC) score as the sum of the differentiation, mitotic count and necrosis score was calculated, then the correspondent tumor grading was assigned [17].

### 2.3. [18F]FDG PET/CT Imaging and Extraction of Radiomic Features

A combined PET/CT imaging was performed using a Siemens Biograph 16 (Siemens Healthineers). Patients fasted for a minimum of 6 hours before the scan, and glucose levels below 150 mg/dl were required at the time of tracer injection. PET/CT acquisition was performed 60 ± 10 min after intravenous injection of an average dose of 5 MBq/kg (0.14 mci/Kg) of F-18-FDG. A non-contrast-enhanced whole-body CT scan was acquired for anatomic localisation and attenuation correction of PET images. The following parameters were used: 120–140 Kev, 4 mm slice thickness using “CAREDose” software to reduce radiation dose and optimize image quality. PET data were acquired on a 3D mode immediately after the CT scan, 2–3 minutes for each bed position. PET images were reconstructed by an ordered subset expectation maximization (OSEM) algorithm (TrueX, Siemens Healthineers) with point spread function modelling (3 iterations, 21 subsets). After reconstruction, the images were filtered by a Gaussian filter with a full width at half maximum of 4 mm. FDG PET/CT data images were reviewed by a nuclear medicine physician with more than 10 years of clinical experience in sarcoma imaging using a dedicated workstation (Syngo.via, Siemens Healthineers). Radiomic features were extracted from PET images using the free software LIFEx v6.3 (Local Image Feature Extraction, www.lifexsoft.org) [18] by two experienced nuclear medicine physicians, who had knowledge of patient clinical data at the time of the PET/CT scan, but who were blinded to patient clinical outcome. A volume of interest (VOI) was contoured manually over the tumor and a semi-automatic method was used for segmentation based on a threshold of SUV 2.5. Voxel intensity was resampled with 64 grey levels and normalized by absolute resampling bounds between 0 and 35 SUV units. Voxels with an SUV greater than 35 were grouped in the highest bin. A total of 45 features were extracted for each VOI. The 14 first-order features were: 4 features from shape (including MTV), 5 from histogram and 5 from conventional indexes (SUVmin, SUVmean, SUVmax, SUVpeak and TLG). The 31 second-order textural features were: 6 from the grey level co-occurrence matrix (GLCM), 11 from the grey-level run length matrix (GLRLM), 3 from the neighbourhood grey-level different matrix (NGLDM) and 11 from the grey-level zone length matrix (GLZLM). A detailed feature description is available in the Texture User Guide of LIFEx (www.lifexsoft.org).

### 2.4. Statistical Analysis

Statistical analyses were performed by R software (ver. 4.1.0, The R Foundation for Statistical Computing, Vienna, Austria). Correlation was evaluated with the Pearson r coefficient for quantitative variables and with Kendal τ in the presence of qualitative ordinal variables. All the clinical (including age and gender), tumor (including size, FNCLC score, grading and other histologic variables described above) and treatment-related parameters already known to affect DFS and OS were analysed [19], except for tumor depth and margins, because 48 out of 51 tumors in the series were deeply located and were all excised with adequate margins. A univariate Cox proportional-hazards regression analysis was performed to evaluate associations between clinical and PET metrics with clinical outcomes (DFS and OS). Statistically significant quantitative parameters were dichotomized for survival analysis using the Maximally selected rank statistics (Package ‘maxstat’ in R). A multivariate Cox proportional-hazard model was then created using stepwise selection of statistically significant (*p* < 0.05) variables in the univariate model. DFS and OS curves were analysed by the Kaplan–Meier method and tested for statistical significance using the log-rank test.

## 3. Results

### 3.1. Clinical Characteristics, PET Parameters and Histologic Features

The study population included 31 males and 20 females, median age 62.4 years. A total of 41 out of 51 patients received peri-operative treatment (neoadjuvant in 7 cases, adjuvant in 25 and neoadjuvant + adjuvant in 9). Sites of primary tumors included the limbs (*n* = 44) or trunk (*n* = 7). All but one patient were classified as stage III (IIIa *n* = 26; IIIb *n* = 24). Detailed patient demographics and clinical characteristics are summarized in Table 1. All tumors showed a significantly high and variable degree of FDG uptake, with a mean SUVmax of 19.2 ± 11.5 (range 4.3–49.4). The median MTV and TLG were 181 cm^3^ (interquartile range [IQR] 60–453 cm^3^) and 1011 (IQR 345–2654), respectively. As expected for this histotype, most histologic specimens showed high numbers of mitoses (many of them atypical), apoptotic figures, high cellularity and pleomorphism, moderate necrosis and low/absent inflammatory infiltrate. Detailed results are summarized in Table 2.

### 3.2. Relationship between Histopathological Data and PET Parameters

**Figure 2:**
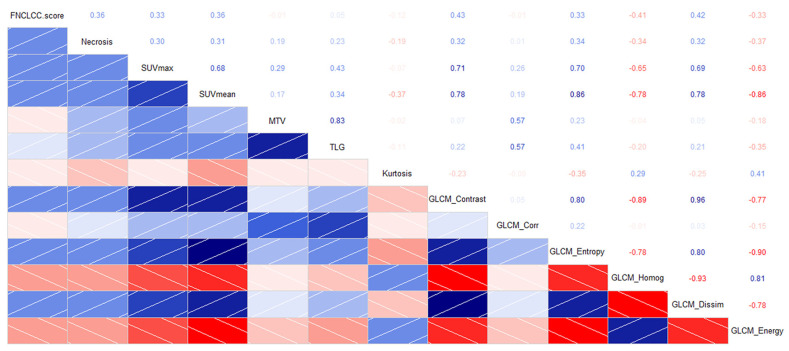
Correlogram including histologic, conventional PET metrics, first order and GCLM textural indices (lower left; blue = direct correlation; red = inverse correlation) with Kendall τ coefficients (upper right).

An inter-correlation among some standard PET parameters and textural indices was observed, as reported in previous studies [20]. The FNCLCC score showed a significant correlation with GLCM_contrast (τ = 0.48; *p* < 0.0001), GLCM_dissimilarity (τ = 0.48; *p* < 0.0001) and GLCM_homogeneity (τ = −0.49; *p* < 0.0001), and to a lesser extent, with SUV indices (SUVmax, SUVmean, SUVpeak), while it was not correlated to PET volumetric parameters (MTV and TLG). Among the histologic parameters analysed, only necrosis was correlated with some PET and textural indices. A correlogram of the most representative histologic, PET and textural indices are represented in Figure 2. 

### 3.3. Disease-Free Survival (DFS)

**Figure 3:**
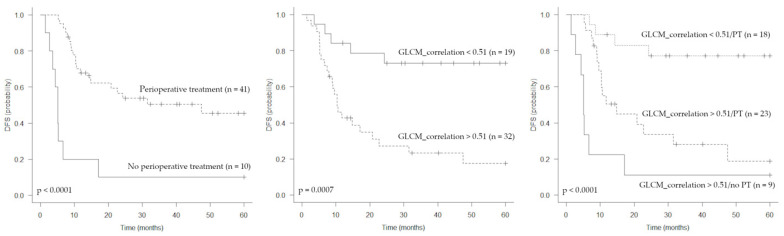
Kaplan–Meier estimations of disease-free survival (DFS) according to perioperative treatment, GLCM_correlation and both parameters combined.

The median follow-up time for all patients was 50.7 months (IQR 30.3–79.8). During the observation period, disease relapse occurred in 29 patients. Distant metastases were observed in 24 patients and local recurrence in 4 patients. One patient experienced both local and distant progression. As expected, lung metastases were the most frequent site of disease progression, occurring in 20 patients. Other metastatic sites were bone, soft tissues and lymph nodes. The median DFS was 20.7 months (95% CI: 10.4 months-not reached). At the univariate analysis, MTV, TLG, GLCM_correlation and perioperative treatment (both chemotherapy and radiotherapy) were associated with DFS (Table 3, Figure 3). All clinical and tumor parameters analysed were not associated to DFS. The GLCM_correlation was positively correlated with both MTV (r = 0.63, *p* < 0.001) and TLG (r = 0.51, *p* < 0.001). Given the expected high degree of correlation between PET volumetric parameters (r = 0.84; *p* < 0.0001), only MTV was considered in the multivariate analysis. At the multivariate analysis, only GLCM_correlation (cut off > 0.51) and perioperative treatment remained prognostic for DFS (Table 3, Figure 3). After disease progression, 21 patients received systemic chemotherapy for the onset of distant metastasis (except in 1 case with multifocal local relapse), 5 patients were submitted to surgery (3 patients for local relapse and 2 for lung metastases), and 1 patient received stereotactic body radiation therapy for lung metastases. Finally, two patients who experienced a rapid disease progression with impairment of performance status were admitted to palliative care.

### 3.4. Overall Survival (OS)

**Figure 4:**
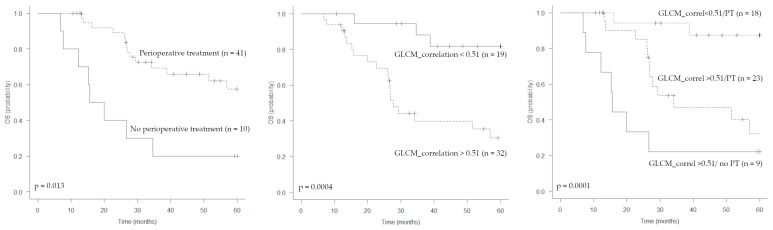
Kaplan–Meier estimations of overall survival (OS) according to perioperative treatment, GLCM_correlation and both parameters combined.

In total, 23 out of 51 patients died during the observation period. The median OS was 56.9 months (95% CI: 29.4-not reached), with a survival rate of 96.1% at 1 year and 58.6% at 3 years. At the univariate analysis, tumor dimension (>10 cm), MTV, GLCM_correlation and perioperative treatment were associated with OS (Table 3, Figure 4). When considered separately, perioperative chemotherapy bordered on statistical significance (*p* = 0.175). By combining the parameter GLCM_correlation with perioperative treatment (Figure 4), patients can be stratified into three different prognostic classes (*p* = 0.0001): (1) low GLCM_correlation/perioperative treatment (survival rate at 3 years 94%, median OS not reached); (2) high GLCM_correlation/perioperative treatment (survival rate at 3 years 46.7%, median OS 34.2 months); (3) high GLCM_correlation/no perioperative treatment (survival rate at 3 years 22.2%, median OS 15.6 months). Only one in ten patients not undergoing any perioperative treatment showed a GLCM_correlation value below the cut off, hence the behaviour of this group cannot be predicted.

## 4. Discussion

The present study aimed to assess the possible prognostic value of conventional PET metrics and textural features extracted from a pre-treatment [18F]FDG PET/CT scan in patients with locally advanced USTS. To the best of the authors’ knowledge, this is the first study focusing on the use of a radiomic approach in STS dealing with a homogeneous histotype class. The key finding of this retrospective study was that the radiomic parameter GLCM_correlation at baseline PET/CT was significantly associated with DFS and OS.

Several previous reports [9,10,12,13,14] analysed the correlation of standard PET parameters (SUVmax, MTV and TLG) with DFS and OS, but all of them dealt with mixed sarcoma histotypes, sometimes also including benign lesions and bone tumors. Indeed, because of the rarity of each histotype, a histology-specific analysis is difficult to perform in both retrospective and prospective studies. In a retrospective series of 55 patients with STS, SUVmax (HR = 1.274, *p* = 0.015), treatment modality (HR = 3.353, *p* = 0.019) and necrosis (HR = 5.985, *p* = 0.006) were identified as significant independent prognostic factors for overall survival [10]. SUVmax was also reported in other studies as a prognostic factor [9,13]. In our series, SUVmax was not significantly predictive of DFS and OS, even at the univariate analysis. SUVmax represents the maximum intensity of a single pixel in the tumor; thus, it does not reflect important properties, such as heterogeneity, size and tumor burden. For this reason, the use of volume-based [18F]FDG metabolic markers have been introduced, and several studies reported a significant prognostic role in many solid tumors [21,22,23], although data on STS are conflicting. A study by Choi et al. on 66 patients with STS reported that TLG is a more accurate predictor of disease progression than SUVmax or MTV [12]. Conversely, in the study by Hong described above, volumetric PET volumes underperformed the prognostic value of SUVmax [10]. In the present study, MTV and TLG showed a prognostic role for DFS and OS only at univariate analysis. Conversely, in the multivariate analysis, only the radiomic parameter GLCM_correlation and perioperative treatment remained prognostic for patient outcome, although chemotherapy was not statistically significant for OS. Even if this data must be considered prudently in relation to the small sample size, it nevertheless appears to be in agreement with the current literature. In fact, while radiotherapy is considered a standard treatment in high-grade STS [4,24], there is no uniform consensus on the use of perioperative chemotherapy, which is often offered in patients at high risk of death [16]. The application of texture analysis on [18F]FDG PET/CT PET/CT, which can facilitate detecting features and patterns not visible to the human eye, is increasing over time. Many studies have shown a significant prognostic role for tumor heterogeneity evaluated from texture analysis of several solid tumors [21,25,26,27,28]. On PET imaging, texture parameters may be related to the cellular and molecular characteristics of the tumor, such as fibrosis, hypoxia and metabolism.

The grey-level co-occurrence matrix (GLCM) characterizes the texture of an image by calculating how often pairs of pixels with specific values and in a specified spatial relationship occur in an image [29]. The FNCLCC score, which represents a histologic surrogate for tumor aggressiveness, showed a significant positive correlation with GLCM_contrast and GCLM_dissimilarity and an inverse correlation with GLCM_homogeneity, all features associated with tumor heterogeneity. Indeed, according to the formula, the first two parameters increase as the grey-level differences between a reference pixel and the neighbouring pixels increase, while the GLCM_homogeneity has an opposite behaviour [29]. Surprisingly, all these parameters were not associated with DFS and OS.

The GLCM_correlation represents the linear dependency of grey-levels in GLCM. The correlation between pixels means that there is a predictable and linear relationship between the two neighbouring pixels within the window, as expressed by the regression equation. GLCM_correlation was previously shown to be an independent prognostic factor in a cohort of 284 patients with head and neck squamous cell carcinomas [25], with a significantly higher OS rate for those with a value <0.14 (57.6% vs. 32.4% at 2 years, *p* < 0.0001). As shown in other studies [20,25], we found a moderate positive correlation of GLCM_correlation with MTV and TLG values. Volumetric PET parameters showed a prognostic role for DFS and OS (only MTV) at the univariate analysis, while GLCM_correlation retains its prognostic value, even at the multivariate analysis, suggesting that it can bring additional information on tumor biology compared to standard volumetric parameters. Furthermore, when we analysed the textural data of nine patients with metastatic USTS at time of diagnosis (not included in this series) separately, we observed that all of them had a GLCM_correlation value above the threshold (median 0.63 ± 0.09 standard deviation), confirming that this parameter could actually be correlated with biological tumor aggressiveness. GLCM_correlation did not show significant correlation with histologic features or with other GLCM textural features (Figure 2). Analysis of survival curves revealed that GLCM_correlation is the most important parameter correlated with OS. As shown in Figure 4, patients who received a perioperative treatment with high values had a significantly worse prognosis than those with low values (survival rate at 3 years 46.7 vs. 94%, median 34.2 months vs. not reached). As expected, the prognosis is even worse in patients with GLCM_correlation above the cut-off, who did not receive any perioperative treatment (survival rate at 3 years 22.2%, median OS 15.6 months). The large tumor size (>10 cm, stage IIIb) is the most widely reported negative prognostic factor for USTS [19,30,31]. In this study, the parameter is significant only for OS at the univariate analysis, probably due to the small population size. In this series, it was not possible to include two other important prognostic parameters in the analysis, namely, tumor depth (a deep lesion was found in 48/51 patients) and surgical margins (adequate in all the specimens).

This study has several limitations. First, the sample size was relatively small, although in accordance with the rarity of the neoplasm. Second, heterogeneous treatment modalities, both in the perioperative setting and after the appearance of local relapse or distant metastasis, may have influenced the survival. This aspect was partially overcome by applying the multivariate analysis. Finally, the retrospective design of the study may have led to a selection bias. Conversely, the strength of the study is that only a specific histotype of STS, homogeneous by stage (IIIa/b in 50/51), was analysed. In fact, it is known that the histologic subtype had a significant impact on patient outcome [32], and that SUVmax is highly variable, even among the high-grade histotypes [7,33]. 

In conclusion, the results presented herein showed that among the PET metrics and textural indices investigated, only GLCM_correlation calculated on pre-treatment [18F]FDG PET/CT PET/CT has a prognostic value in patients with resectable USTS. If the prognostic value of this feature is confirmed in prospective studies with larger populations, it could be proposed to select high-risk patients who can better benefit from combined neoadjuvant and/or adjuvant treatment.

## Figures and Tables

**Table 1:** Patient demographics and clinical characteristics.

Characteristics	Data
Age (years), median (IQR)	62 (54–72)
Gender	
Male Female	31 (60.8%) 20 (39.2%)
Site of tumor	
Lower limb Upper limb trunk	*n* = 43 (84.3%) *n* = 1 (1.9%) *n* = 7 (13.7%)
Tumor size (cm), median (IQR)	10 (6.8–16.4)
Tumor depth	
Deep Superficial	*n* = 48 (94.1%) *n* = 3 (5.9%)
AJCC stage	
II (<5 cm) IIIa (>5 and <10 cm) IIIb (>10 cm)	*n* = 1 (2%) *n* = 26 (51%) *n* = 24 (47%)
Neoadjuvant treatment Chemotherapy Radiotherapy	*n* = 16 (31.4%) *n* = 15 (29.4%) *n* = 1 (2%)
Adjuvant treatment Radiotherapy alone radiotherapy+chemotherapy chemotherapy alone	*n* = 33 (64.7%) *n* = 13 (25.5%) *n* = 12 (23.5%) *n* = 8 (15.7%)

IQR, Interquantile range.

**Table 2:** Histologic features.

Characteristics	Data
Histologic grade	
G2 G3	*n* = 11 (21.6%) *n* = 40 (78.4%)
Histology subtype	
Pleomorphic Spindle cell	*n* = 44 (86.3%) *n* = 7 (13.7%)
Mitoses/10 HPF median (IQR)	18 (11–28)
Atypical mitoses	
yes no	*n* = 38 (74.5%) *n* = 13 (25.5%)
Apoptosis	
absent rare diffuse	*n* = 18 (35.3%) *n* = 24 (47.1%) *n* = 9 (17.6%)
Pleomorphism	
mild moderate severe	*n* = 18 (35.3%) *n* = 22 (43.2%) *n* = 11 (21.6%)
Necrosis (%) median (IQR)	20 (5–30)
Cellularity (%) median (IQR)	70 (52–75)
Inflammatory infiltrate	
low/absent high	*n* = 37 (72.5%) *n* = 14 (27.5%)

HPF, High Power Field; IQR, Interquantile range.

**Table 3:** Univariate and multivariate Cox proportional-hazards regression for the prediction of DFS and OS.

Univariate Analysis	DFS		OS	
Parameter	*HR (95% CI)*	*P*	*HR (95% CI)*	*P*
Gender (male)	1.5 (0.7–3.3)	0.31	1.3 (0.6–3)	0.55
Age Age > 60 years	1.02 (0.99–1.05) 1.46 (0.7–3.2)	0.28 0.33	1.0 (0.98–1.0) 1.2 (0.5–2.8)	0.75 0.69
Tumor dimension Tumor dimension > 10 cm	0.98 (0.93–1.03) 2.06 (0.97–4.4)	0.38 0.06	0.98 (0.93–1.04) 2.53 (1.07–6)	0.51 **0.035**
Perioperative treatment (any)	0.19 (0.08–0.42)	**<0.001**	0.27 (0.11–0.63)	**0.03**
MTV > 89 cm^3^	3.5 (1.3–9.1)	**0.012**	3.4 (1.1–9.9)	**0.03**
TLG > 280	5.9 (1.4–24.8)	**0.016**	3.9 (0.9–16.6)	**0.07**
GLCM__corr_ > 0.51	4.6 (1.8–12.2)	**0.002**	6.7 (1.9–22.6)	**0.002**
Multivariate analysis				
Perioperative treatment (any)	0.29 (0.13–0.68)	**0.004**	0.40 (0.17–0.96)	**0.04**
GLCM__corr_ > 0.51	3.4 (1.25–9.5)	**0.017**	5.5 (1.59–19.1)	**0.007**

Bold *P* Values indicate significant differences.

## Data Availability

The data presented in this study are available on request from the corresponding author.
